# Supplementary material for: Causal Relationship between Aging and Anorexia Nervosa: A White-Matter-Microstructure-Mediated Mendelian Randomization Analysis
Source: Biomedicines. 2024 Aug 16;12(8):1874. doi: 10.3390/biomedicines12081874 (PMC11351342; doi:10.3390/biomedicines12081874)
Supplement: Supplementary file 1 [file biomedicines-12-01874-s001.zip › Supplementary Figure/Figure S4/Singe SNP plot of MR analysis for TL to ubm-b-1865.pdf]

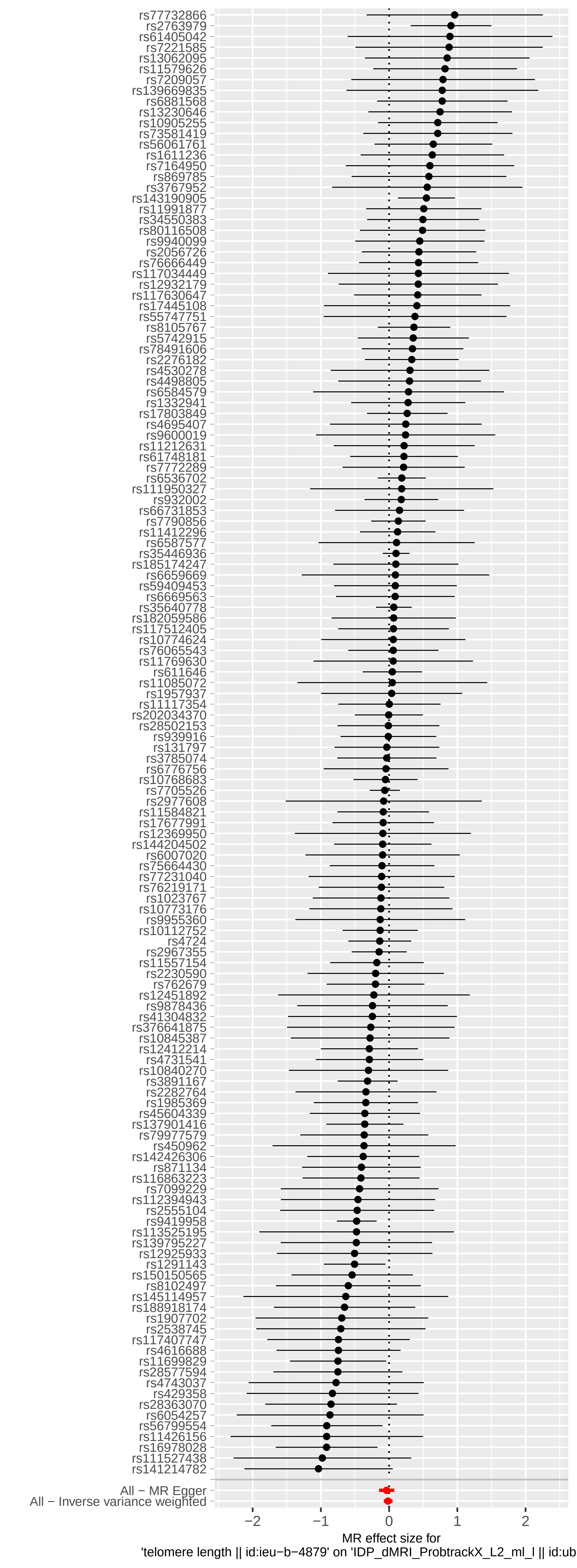
rs77732866
rs2763979
rs61405042
rs7221585
rs13062095
rs11579626
rs7209057
rs139669835
rs6881568
rs13230646
rs10905255
rs73581419
rs56061761
rs1611236
rs7164950
rs869785
rs3767952
rs143190905
rs11991877
rs34550383
rs80116508
rs9940099
rs2056726
rs76666449
rs117034449
rs12932179
rs117630647
rs17445108
rs55747751
rs8105767
rs5742915
rs78491606
rs2276182
rs4530278
rs4498805
rs6584579
rs1332941
rs17803849
rs4695407
rs9600019
rs11212631
rs61748181
rs7772289
rs6536702
rs111950327
rs932002
rs66731853
rs7790856
rs11412296
rs6587577
rs35446936
rs185174247
rs6659669
rs59409453
rs6669563
rs35640778
rs182059586
rs117512405
rs10774624
rs76065543
rs11769630
rs611646
rs11085072
rs1957937
rs11117354
rs202034370
rs28502153
rs939916
rs131797
rs3785074
rs6776756
rs10768683
rs7705526
rs2977608
rs11584821
rs17677991
rs12369950
rs144204502
rs6007020
rs75664430
rs77231040
rs76219171
rs1023767
rs10773176
rs9955360
rs10112752
rs4724
rs2967355
rs11557154
rs2230590
rs762679
rs12451892
rs9878436
rs41304832
rs376641875
rs10845387
rs12412214
rs4731541
rs10840270
rs3891167
rs2282764
rs1985369
rs45604339
rs137901416
rs79977579
rs450962
rs142426306
rs871134
rs116863223
rs7099229
rs112394943
rs2555104
rs9419958
rs113525195
rs139795227
rs12925933
rs1291143
rs150150565
rs8102497
rs145114957
rs188918174
rs1907702
rs2538745
rs117407747
rs4616688
rs11699829
rs28577594
rs4743037
rs429358
rs28363070
rs6054257
rs56799554
rs11426156
rs16978028
rs111527438
rs141214782
All − MR Egger
All − Inverse variance weighted
−2
−1
0
1
2
MR effect size for
'telomere length || id:ieu−b−4879' on 'IDP_dMRI_ProbtrackX_L2_ml_l || id:ub
